# Evaluation of cosmetic appearance of herniotomy wound scars in African children: Comparison of tissue glue and subcuticular suturing

**DOI:** 10.4103/0970-0358.59282

**Published:** 2009

**Authors:** A. O. Ademuyiwa, O. A. Sowande, O. Adejuyigbe, U. E. Usang, T. I. B. Bakare, L. J. C. Anyanwu

**Affiliations:** Paediatric Surgery Unit, Department of Surgery, Obafemi Awolowo University Teaching Hospitals' Complex, Ile Ife, Osun State, Nigeria

**Keywords:** Scars on African skin, tissue glue, wound healing in Africans

## Abstract

**Aim:**

To evaluate the cosmetic appearance of herniotomy wound scars closed using either the tissue glue or subcuticular suturing technique.

**Materials and Methods:**

Prospective randomised control study; randomisation into tissue glue and suturing groups. Ethical clearance obtained. Cosmetic outcome were based on visual analogue scale by parents and Hollander wound evaluation scale by a Plastic Surgeon blinded to the wound closure method.

**Results:**

Fifty one wounds were evaluated, 26 in the tissue glue group and 25 in the suturing group. Parents' evaluation using Visual Analogue scale (VAS) showed that in the suturing group, 17 parents (68%) gave a VAS of 8cm while six parents (24%) gave a score of 7cm. Two parents (8%) gave a score of 9cm. In the tissue glue group, 22 parents (84.6%) scored the scar of their children as 8 or 9cm on the VAS while four parents (15.4%) gave a score of 7cm. The median VAS was 8cm for both groups with a range of 7 to 9cm. The Chi- square test showed that the parents preferred tissue glue compared with subcuticular suturing (X2 = 7.90, P < 0.05). The Hollander Wound Evaluation Scale (HWES) used by Plastic Surgeon showed 21 herniotomy wounds (84%) had a score of 6 in the suturing group while four wounds (16%) had a score of 5. In the tissue glue group, 19 wounds (73%) had a score of 6, six wounds (23.1%) had a score of 5 and a patient (3.8%) had a score of 4. The median score is 6 for both groups. There was no statistically significant difference between both groups (X^2^ = 1.481, P = 0.393).

**Conclusion:**

This study has shown that the cosmetic outcome of wound closure using the tissue glue technique and subcuticular suturing technique are similar.

## INTRODUCTION

Wound closure is an essential part of surgery. In the past, insect tick, flax and animal fibres has been used to close wounds.[1] Technological advancement has made better materials for wound closure possible including absorbable and non absorbable sutures, tapes, zippers and glues.[2,3]

The tissue glue (octylcyanoacrylate) has been widely studied and is said to confer some advantages including better cosmetic outcome, faster wound closure time as well as less wound edge related complications.[2,4–6] It also has the added advantage of not requiring removal of sutures postoperatively.

In our practice, the subcuticular suturing technique is used to close herniotomy wounds because of its cosmetic outcome as well as the fact that there is no need to remove the sutures following surgery. The use of tissue glue is relatively new in our centre, and a few studies from Africa have investigated the cosmetic outcome of its use. We conducted a prospective randomised study to evaluate the cosmetic outcome of herniotomy wounds using the tissue glue compared with the subcuticular suturing technique.

## MATERIAL AND METHODS

This is a prospective randomised controlled study carried out in the Paediatric Surgery Unit of the Obafemi Awolowo University Teaching Hospitals Complex (OAUTHC) Ile Ife, south west of Nigeria. The surgical procedures for this study were done at the Day Case Surgical Centre of the hospital.

Patients were recruited into the study from those seen at the Paediatric Surgical Outpatient (PSOP) clinic presenting with inguinal hernias. Selection was based on a stratified randomised sampling technique. Patients were stratified into different age groups with similar physiological characteristics and psychosocial development. The different groups included: Infancy, 1 – 5years, 6 - 10 years, and 11 – 15 years. Patients falling into each group were thereafter randomly chosen by tossing a coin with ‘head’ representing the suture group while ‘tail’ represented the tissue glue group. For patients with bilateral hernia, however, both wounds were grouped into same group because many of the parents preferred that one of the methods should be used for their children and not both methods on the same child.

Fifty two wounds were recruited into the study with 26 each in study and control group 26. The suture used for patients recruited into the suture wound closure group was the 0000 polyglycolic acid (Dexon®) sutures. The tissue glue 2-Octylcyanoacrylate (Dermabond® manufactured by Ethicon) was obtained from commercial retailers. The study was commenced after due clearance from the Ethics and Research Committee of the Hospital.

Herniotomy through a skin crease incision was performed in all the patients with high ligation of the hernial sac at the internal inguinal ring.[7] The wounds were closed in layers i.e. the external oblique aponeurosis followed by the superficial fascia, with absorbable sutures in both groups. The apposition of the skin was with 2 - octylcyanoacrylate in the study group while it was with subcuticular suturing technique in the control group. The application of 2-octylcyanoacrylate was done after the superficial fascia of Camper's has been properly apposed. Toothed dissecting forceps were used to appose the wound about 1cm from its medial and lateral limits leaving no space between the apposing wound edges. The tissue glue was then applied carefully on the skin surface making sure that the glue did not seep in between the wound edges. Special precautions as directed by the manufacturers were explained to the parents for strict compliance. Wounds were reviewed for wound edge complications on the 4^th^ and 7^th^ postoperative day as well as 4^th^, 8^th^ and 12^th^ weeks postoperatively.

Patients in the subcuticular suturing wound closure group had wound dressings with isotonic saline solution and methylated spirit on the fourth post operative day with gauze dressings while by the seventh day post operation, open dressing with methylated spirit only was performed. Patients in the Dermabond® wound closure group had their wounds inspected with change of gauze only on the fourth postoperative day as the thin film of the tissue glue remained and usually fell off within 7 – 14 days. They did not require any dressing solutions.

Assessment of cosmetic appearance based on the Hollander Wound Evaluation Scale and Visual Analogue Scale[2,8,9] was done by the Plastic Surgeon (who was blinded to the method of wound closure) and the parents respectively. The assessment was based on parents' satisfaction with the wound using the Visual Analogue Scale (A sheet of paper on which a 10 – centimetre line is drawn and divided into 10 equal parts from which the parents were asked to assess their level of satisfaction). Zero represented the worst scar the parents could ever imagine while 10 represented the finest scar possible. A Plastic Surgeon assessed the wound using the Hollander Wound Evaluation Scale. These assessments were done at four weeks and 12 weeks post surgery. Also, any sign of allergy to the tissue glue were sought and documented if found.

The data collated was analysed using the Statistical Package for the Social Sciences (SPSS) version13.0. The Chi-square test was used to test the statistical significance of the results; a p value of < 0.05 was taken to be significant.

## RESULTS

There were 52 wounds from 45 patients, seven of whom had bilateral inguinal hernias. However, only 51 wounds were evaluated. A patient in the subcuticular suturing group with surgical site infection was excluded from the study as this could affect the eventual outcome of the scar.

Of the 37 who did not have bilateral hernias, 21 were randomised into the subcuticular suturing group while 16 were in the tissue glue group. There were 26 wounds in the tissue glue method of wound closure and there were 25 wounds in the subcuticular suturing method of wound closure. There was only one female who presented during the study period.

Thirty patients (58.8%) were in the age group of one to five years while 11 (21.6%) were between six and 10 years of age. There were five patients (9.8%) each in the infant group and those between 11 and 15 years [Table 1]. There were 20 (45.5%) patients with right inguinoscrotal hernias while 17 (38.6%) patients had left inguinoscrotal hernias. Seven patients had bilateral hernias representing 15.9% of the patient population.

**Table 1:** Frequency distribution age of patients and the method of wound closure

*Age of patients*	*Method of wound closure*		*Total (%)*
		
	*Dermabond*	*Suture*	
0-12mths	3	3	6 (11.5)
1- 5yrs	12	18	30 (57.7)
6- 10yrs	8	3	11 (21.2)
11-15yrs	3	2	5 (9.6)
Total	26	26	52 (100.0)

There were two cases of erythema around the wound edges complicating the wound closure (one in tissue glue group and another in the subcuticular suturing group). They resolved spontaneously within a week of surgery. In the subcuticular suturing group, 17 parents (68%) gave a Visual Analogue Scale (VAS) score of 8cm while six parents (24%) gave a score of 7cm. Two parents (8%) gave a score of 9cm [Figure 1A and 2A].

**Figure 1A:**
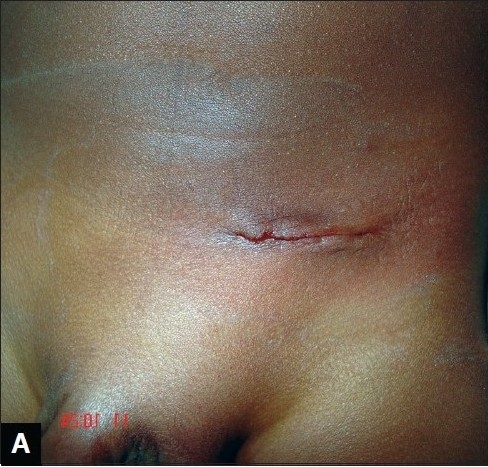
Perioperative appearance of herniotomy scar (subcuticular suturing technique)

**Figure 2A:**
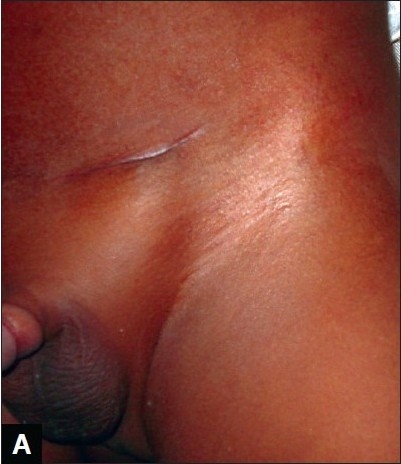
Appearance of herniotomy scar at 12 weeks (subcuticular suturing technique)

Twenty two parents (84.6%) whose children had their wounds closed with octylcyanoacrylate scored the scar of their children [Figure 1B and 2B] as 8 or 9cm on the Visual Analogue Scale while 4 parents (15.4%) gave a score of 7cm [Table 2]. The median VAS was 8cm for both groups with a range of 7 to 9cm. The Chi square test showed that the parents preferred tissue glue compared with subcuticular suturing (X^2^ = 7.90, p < 0.05).

**Figure 1B:**
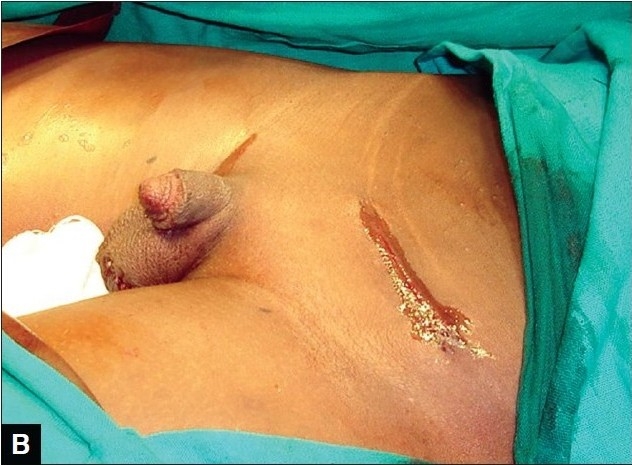
Perioperative appearance of herniotomy scar (tissue glue technique)

**Figure 2B:**
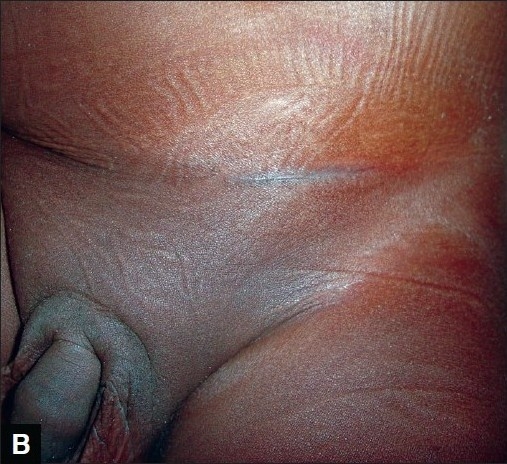
Appearance of herniotomy scar (tissue glue technique)

**Table 2:** Showing the parental visual analogue scale according to wound closure method

*Visual analogue scale (parents)*	*Dermabond wound closure*	*Subcuticular wound closure*	*Total*
4	0	1	1
7	4	6	10
8	11	17	28
9	11	2	13
Total	26	26	52

With the use of Hollander Wound Evaluation Scale (HWES) by the Plastic Surgeon, 21 herniotomy wounds (84%) had a score of 6 in the Subcuticular suturing group while 4 wounds (16%) had a score of 5. In the study group closed with 2 – octylcyanoacrylate, 19 wounds (73%) had a score of 6, six wounds (23.1%) had a score of 5 and a patient (3.8%) had a score of 4 [Table 3]. The median score was 6 for both groups. There was no statistically significant difference between both groups (X^2^ = 1.481, p = 0.393).

**Table 3:** Hollander Wound Evaluation Scale score by Plastic Surgeon according to wound closure method

*Hollander Wound Evaluation Scale Score (0 – 6)*	*Dermabond wound closure*	*Subcuticular wound closure*	*Total*
3	0	1	1
4	1	0	1
5	6	4	10
6	19	21	40
Total	26	26	52

## DISCUSSION

The first tissue glue was synthesised in 1949. Since that time, there have been attempts to appose wounds using tissue glues with the possible advantage of shorter wound closure time, less wound related complications such as wound infection and wound dehiscence, less postoperative pain and better cosmetic appearance.[2,4] Several studies suggest that the use of tissue glue in wound closure is as effective as the traditional suturing techniques and some studies actually show that it may be superior to suturing.[10] Most of these studies were done in Western countries and Asia with a few reports from Africa; hence the motivation for this study.

Parents in both groups, tissue glue and subcuticular suturing, were satisfied with the cosmetic outcome of the scars of their children/wards using the Visual Analogue Scale [Figures 1A, 1B, 2A, 2B]. However, it seems parents were more satisfied with use of tissue glue than subcuticular suturing considering that 44% of the parents in the tissue glue group gave a VAS score of 9cm compared with eight per cent of parents whose children's wound were closed using the subcuticular suturing technique. This difference was statistically significant (*P*< 0.05).

The Hollander Wound Evaluation Scale (HWES) is a validated scale[2,11] and after its use, only one wound was unacceptable (having a score <5). The one scar which was unacceptable was in the tissue glue group and had a score of 4. With this objective assessment, however, there was no significant difference in cosmetic outcome between the methods of wound closure. This shows that the cosmetic appearance is comparable irrespective of the method of closure of the wounds.

While this study showed that wound closure with the use of Dermabond has the same cosmetic appearance as the time honoured subcuticular suturing technique using the validated HWES, the use of octylacrylate is considered superior to subcuticular suturing with the use of VAS by the parents. The difference in these two scales may be due to the fact that the VAS may be more subjective than the HWES, especially when specific characteristics are not sought to come to a score. Secondly, it is possible that if a larger sample size were used, this difference may become less apparent.

The satisfaction of parents is a major factor to consider in evaluating the outcome and quality of paediatric surgical practice.

Although this study has shown that the use of tissue glue in African children has at least the same cosmetic appearance when compared with, or may even be superior to the subcuticular suturing technique of wound closure, cost would be a major consideration in the adoption of this method as most of the patients are poor and the cost of closure using tissue glue is about seven times that of closure with suturing technique.

It is to be noted, however, that a limitation of this study is the fact that due to the preference of the parents/guardians of patients with bilateral inguinal hernias not to have both types of wound closure methods on their children and wards, the individual wounds could not be randomised. In addition, each of such parents had to vote twice. Further studies with larger sample sizes are necessary to confirm or refute some of our observations.
